# Swiss Cheese Flavor Variability Based on Correlations of Volatile Flavor Compounds, Descriptive Sensory Attributes, and Consumer Preference

**DOI:** 10.3390/foods8020078

**Published:** 2019-02-19

**Authors:** Hardy Z. Castada, Kaitlyn Hanas, Sheryl Ann Barringer

**Affiliations:** Department of Food Science and Technology, The Ohio State University, 2015 Fyffe Road, Columbus, OH 43210, USA; taylor.2121@buckeyemail.osu.edu

**Keywords:** Swiss cheese flavor, selected ion flow tube-mass spectrometry (SIFT-MS), descriptive sensory analysis, odor activity values (OAVs)

## Abstract

Minimizing flavor variation in cheeses without perceived flavor defects in order to produce a consistent product is a challenge in the Swiss cheese industry. This study evaluated flavor variability based on correlations of volatile flavor compounds and sensory attributes. The headspace concentrations of volatile compounds were analyzed using selected ion flow tube-mass spectrometry (SIFT-MS), while the sensory attributes were evaluated using descriptive sensory analysis and consumer testing. The important discriminating volatile compounds were classified into five functional groups: sulfur-containing compounds (methyl mercaptan, hydrogen sulfide, dimethyl disulfide, dimethyl trisulfide, and methional), organic acids (propanoic acid, acetic acid, 3-methylbutanoic acid), aldehydes (3-methylbutanal, butanal, and 2-methylpropanal), a ketone (2,3-butanedione), and an ester (ethyl hexanoate). Correlations were identified among volatile compounds and between volatile compounds and sensory attributes. Only a small number of volatile compounds strongly correlated positively or negatively to a specific sensory attribute. Nutty malty, milkfat lactone, salty, umami, and sweet positively correlated to overall liking and nutty flavor liking of Swiss cheese. Evaluation of cheese flavor using correlations between volatile compounds and sensory attributes provided further understanding of the complexity of flavor and flavor variability among Swiss cheeses manufactured from different factories that can be used to improve flavor consistency of Swiss cheeses.

## 1. Introduction

Flavor consistency is a challenging task for the Swiss cheese industry. Flavor, a fundamental basis for sensory assessment, is an important characteristic that determines consumer choice and acceptance [1,2,3]. The biochemical events occurring during cheese ripening involve complex microbiological and physicochemical changes to the curd that give the characteristic flavor and aroma of a particular cheese variety [4,5,6]. The characteristic nutty, mild, and slightly sweet flavor of Swiss cheese after 60 days or more of curing and ripening becomes more aromatic as the cheese ages with its characteristic eyes formed by CO_2(g)_ [6,7,8]. 

Previously, high impact key volatile compounds were determined to discriminate Swiss cheese samples from different manufacturers using odor activity values (OAVs) [9]. OAV is defined as the concentration of a compound divided by its odor or sensory threshold value. Compounds with concentrations greater than their odor or sensory threshold value are considered to be key, high impact volatiles that significantly contribute to flavor [9,10]. To further understand the differentiation and flavor variability in Swiss cheese, it is important to study the compounds affecting the behavior (i.e., intensity) of a particular flavor compound. In reference to the component balance theory, the flavor of cheese results from a balanced concentration and mixture of a wide variety of volatile and non-volatile flavor compounds [11,12,13]. Because of this mixture of compounds, it is useful to study the correlation of compounds especially to key flavor volatiles and understand the physicochemical effect they have on each other. An increase or suppression of volatile intensity or even generation of new aroma qualities of odorants may change by interaction, causing changes in the overall flavor profile [14,15,16,17]. 

One of the ultimate goals in flavor chemistry research is to identify the relevance and association of flavor compounds with sensory perceptions and attributes. However, finding and interpreting these associations and interactions are difficult and challenging because the basis of their methodologies is fundamentally different. Nonetheless, there is a much-improved understanding and better appreciation of flavor when a specific correlation between volatile compounds and sensory attributes is established, which is especially useful for resolving flavor issues [18,19,20,21].

The objective of this study was to further understand flavor variability in Swiss cheese samples without perceived flavor defects, in relation to volatile flavor compounds and sensory attributes. The concentrations of Swiss cheese volatile flavor compounds and the intensities of sensory attributes were measured. The correlations among the volatile flavor compounds and sensory attributes were then evaluated. Descriptive analysis and consumer testing were performed to understand flavor characteristics and attributes that affect consumer liking and the overall consumer preferences that determine flavor variation in Swiss cheese.

## 2. Materials and Methods 

### 2.1. Swiss Cheese Volatile Flavor Compound and Odor Activity Value (OAV) Analyses 

Swiss cheese blocks (8–10 lb (3.6–4.5 kg) cheese block) were obtained from five different factories (148, 207, 374, 465, and 528) and were vacuum-packed and stored at −40 °C if not immediately utilized. Table 1 lists the ages of the Swiss cheese samples and the different starter and adjunct cultures used by each factory. 

For headspace volatile compound analysis, shredded Swiss cheese samples (5 g) were placed into a 500-mL Schott bottle and capped with a polytetrafluoroethylene (PTFE)-lined silicone septum. The samples were warmed for 1 h at 40 °C to allow headspace equilibration prior to volatile analysis using selected ion flow tube-mass spectrometry (SIFT-MS, V200 Syft Technologies, Christchurch, New Zealand). Immediately after heating, the headspace sampling was carried out by inserting a passivated sampling needle (~3.5 cm) through the bottle’s septum. The sample inlet flow rate was 0.28 ± 0.01 Torr•L s^−1^ (21 ± 1 cm^3^ min^−1^) under standard ambient temperature (298 K). A selected ion monitoring (SIM) scan method was used to measure the headspace concentration of volatile flavor compounds for a total scan duration of 120 s. Table 2 lists the volatile compounds and their corresponding kinetic information used within the SIM scan method for volatile compound analysis [22]. 

In SIFT-MS, volatiles are identified and quantified via known product ions resulting from the soft chemical ionization of the selected compounds with the instrument’s reagent ions (H_3_O^+^, NO^+^, and O_2_^+^). The headspace volatile compound concentration is obtained using the reaction-specific rate constant (*k*), reaction time (*t*_r_), and the count rates of the reagent ions ([R^+^]) and the product ion ([M^+^]) [23,24,25]. Subsequently, odor activity values were obtained by dividing the measured headspace concentration of a volatile compound by its odor threshold value [7,9,26]. The odor threshold values in air were obtained from the literature [27]. 

### 2.2. Descriptive Sensory Analysis

Flavor evaluation using descriptive sensory analysis is a powerful sensory tool for cheese flavor research. It involves effective consumer testing to assess cheese flavor acceptability, preference, and consumer perception [28,29]. Although descriptive sensory analysis cannot provide analytical data on specific flavor compounds and flavor profiles, linking instrumental analysis with descriptive sensory analysis could effectively characterize specific flavors significantly influencing consumer acceptability where important flavor attributes are perceived that directly correlate to consumer preferences [28,29,30]. For descriptive sensory analysis, Swiss cheese cut into cubes (2.5 cm^3^) were served at room temperature (25 °C) in a 64-mL translucent plastic soufflé cup labeled with a random three-digit factory code (Table 1).

The analysis was conducted at North Carolina State University. Eight panelists (six females, two males, ages 23–44 years) were trained in the Spectrum^TM^ descriptive analysis method [31,32]. Panelists were trained (10 h each) in specific Swiss cheese flavor attributes prior to cheese assessment to carefully identify and scale Swiss cheese flavor descriptors. Each panelist evaluated three Swiss cheese samples per factory using a previously developed cheese flavor sensory language adapted to Swiss cheese flavor that included 21 attributes (Table 3) [30,33]. For assessment, a 0 to 15-point universal intensity scale was used in accordance with the Spectrum^TM^ method for a uniform scoring of intensities across all cheese attributes [31].

Cheeses were evaluated in a randomized order in booths dedicated to sensory analysis and free from external aromas, noise, and distractions. Compusense Five version 5.2 (Compusense, Guelph, ON, Canada) was used for data collection. Panelists were instructed to expectorate samples after evaluation, and spring water was available to each panelist for palate cleansing. 

### 2.3. Consumer Testing

While descriptive sensory analysis provides information about the sensory aspects of products, effective consumer tests provide information on consumer liking and could give further understanding of the cheese flavor variation from a consumer perspective [31]. For consumer testing, 100 untrained consumers (76 females and 24 males, 18–50 years old) were pre-recruited from the Food Industry Center database at The Ohio State University. Demographics indicated that over 75% of the panelists consumed Swiss cheese regularly or at least twice every two weeks. A cheese sample from each factory was served at room temperature in a 64-mL translucent plastic soufflé cup labeled with the same three-digit codes. All samples were presented in randomized order to reduce potential first order bias. Panelists evaluated samples individually in separate booths and entered their own responses directly into a computer using Compusense Five data collection and analysis software (Compusense^®^ Five, version 5.2, Compusense Inc., Guelph, ON, Canada). Panelists assessed visual characteristics before tasting and rated each sample independently for overall liking and nutty flavor liking on a 9-point vertical hedonic category scale. Panelists were allowed to swallow or expectorate samples, as desired. Sample re-tasting was allowed, and each panelist proceeded at his or her own pace. Room temperature spring water was provided for rinsing between samples.

### 2.4. Statistical Analysis

Multidimensional datasets from volatiles and sensory evaluation were analyzed using principal component analysis (PCA, XLSTAT, Addinsoft, New York, NY, USA) with varimax rotation (clockwise rotation of 15° or cosine of 0.97) [34,35]. Squared cosine (cos^2^) and factor loading values were evaluated to determine the most significant discriminating volatile compounds (i.e., compounds mostly correlated to the principal components (PCs)) and assess volatile correlation between a volatile compound, sensory attribute, or cheese factory (i.e., observation or variable) and a PC axis. A large value of cos^2^ (i.e., cos^2^→1) for an observation or a variable indicates that a PC contributes a relatively large portion of the total distance projected by an observation or variable to the PC. This suggests that a particular observation or variable correlates significantly to the PC and is therefore considered an important observation or variable for discrimination. The factor loading values, on the other hand, determine the variables that account for the differences among observations and thus the correlation (positive or negative) between a component and a variable [35,36]. 

The statistical relationship or association among volatile compounds or sensory attributes, and principal component space was evaluated based on the dimensionless Pearson’s correlation coefficient test statistic (*r*), a method of assessing a possible two-way linear association between two continuous variables and squared cosines [37]. The magnitude and direction of the relationship (−1.00 ≤ *r* ≥ +1.00) indicate the strength and linear association of variables. Correlation between attributes and volatile compounds is described as either strong direct or a strong inverse relationship as *r* approaches (+1) or (−1), respectively. An *r* = 0 indicates negligible correlation between continuous variables. In this study, we adopted a simple rule of thumb for interpreting the size of *r* as follows: 0.90 ≤ *r* ≤ 1.00 or −0.90 ≥ *r* ≥ −1.00 (very high positive or negative correlation, respectively); 0.70 ≤ *r* ≤ 0.89 or −0.70 ≥ *r* ≥ −0.89 (high positive or negative correlation, respectively); 0.50 ≤ *r* ≤ 0.69 or −0.50 ≥ *r* ≥ −0.69 (moderate positive or negative correlation, respectively); 0.30 ≤ *r* ≤ 0.49 or −0.30 ≥ *r* ≥ −0.49 (low positive or negative correlation, respectively); and 0.29 ≥ *r* ≥ −0.29 (negligible correlation) [37,38,39]. 

Correlations between volatile compounds or sensory attributes were evaluated via correlation circle plots using statistical integrative approaches (i.e., partial least squares regression). The correlation between a volatile or a sensory variable and a component are the coordinates (centered and standardized) projected on the PC axes. The angle between two vectors defines the nature of the correlation between two variables. A sharp, acute angle (cos(α) > 0) signifies a positive correlation, while an obtuse or straight angle (cos(β) < 0) indicates a negative correlation, and a right angle (cos(θ) ≈ 0) suggests a null correlation between two volatile compounds or sensory attributes [40,41]. For a more conservative approach, and to further simplify our analyses, the following conditions were applied as thresholds: strong positive correlation (cos(0 ± 8°)): cos(8) > cos(α) > cos(352); strong negative correlation (cos(180 ± 8°)): cos(172) < cos(β) < cos(188); and strong null correlation (cos(90 ± 1°)): cos(89) < cos(θ) < cos(91). A positive correlation indicates that a compound or attribute is well-linked to another compound or attribute, respectively (i.e., parallel increase or decrease in the concentrations of two volatile compounds or intensities of two sensory attributes). A negative correlation suggests that an increase in a volatile concentration or intensity of a sensory attribute causes a decrease in the concentration of another compound or intensity of a sensory attribute, respectively. A null or orthogonal correlation indicates no correlation between two variables, such that an increase or decrease in the volatile concentration of a compound or intensity of a sensory attribute does not affect another volatile compound or sensory attribute.

## 3. Results and Discussion

### 3.1. Variability and Correlation of Swiss Cheese Volatile Compounds

**Figure 1:**
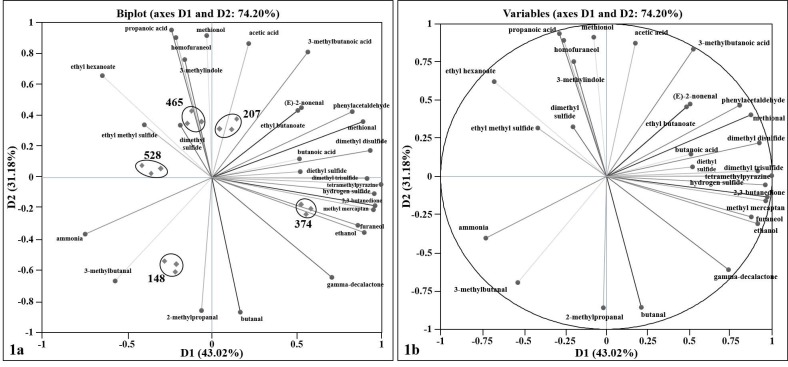
Principal component analysis showing (**1a**) PCA biplot differentiation of Swiss cheeses from different factories (148, 528, 465, 207, and 374) based on volatile compounds and (**1b**) a correlation circle projection of volatile compounds.

The biplot in Figure 1a shows a simultaneous representation of volatile compounds and Swiss cheese samples from different factories in principal component (PC) space. The figure shows clear differentiation of Swiss cheeses by production factory. The differentiation is primarily described by the first two components, which accounted for 74% of the total data variability. The first component (D1) separates factories 465, 528, and 148 from factories 207 and 374. The second component (D2) separates factories 528, 465, and 207 from factories 148 and 374. The significant discriminating volatile compounds are methyl mercaptan, hydrogen sulfide, dimethyl disulfide, dimethyl trisulfide, methional, 2,3-butanedione, ethyl hexanoate, propanoic acid, acetic acid, 3-methylbutanoic acid, 3-methylbutanal, butanal, and 2-methylpropanal. Because the Swiss cheese samples used in this study did not have perceived flavor defects, these volatile compounds are important to distinguish flavor variability between good cheese samples (i.e., cheeses without flavor defects), as companies work to produce a consistent product. 

The discriminating volatile compounds can be classified according to their functional group. The major groups include the sulfur-containing compounds (methyl mercaptan, hydrogen sulfide, dimethyl disulfide, dimethyl trisulfide, and methional), organic acids (propanoic acid, acetic acid, 3-methylbutanoic acid), aldehydes (3-methylbutanal, butanal, and 2-methylpropanal), a ketone (2,3-butanedione), and an ester (ethyl hexanoate). Many of the compounds with the same functional group originate from the same biochemical pathway of production in cheese [8,42,43,44,45]. Inherently, this implies that although the main biochemical pathways during the manufacture and ripening of Swiss cheese remain the same, the activity of some pathways producing volatile compounds could be characteristically more or less dynamic in each factory, causing flavor variation among Swiss cheeses. 

Correlation analysis between volatile compounds (Figure 1b) provided additional insights into the variability of Swiss cheeses from different factories. The discriminating compounds with significantly positive correlations with other compounds (both discriminating and non-discriminating) include 2,3-butanedione with methyl mercaptan and hydrogen sulfide; dimethyl disulfide with butanoic acid and diethyl sulfide; dimethyl trisulfide with tetramethylpyrazine, diethyl sulfide, and hydrogen sulfide; ethyl hexanoate with ethyl methyl sulfide; hydrogen sulfide with methyl mercaptan; methional with phenylacetaldehyde; methyl mercaptan with furaneol; and propanoic acid with 3-methyl indole and homofuraneol. On the other hand, the discriminating compounds with significantly negative correlations with other compounds (both discriminating and non-discriminating) include 2-methylpropanal with 3-methylbutanoic acid, ethyl butanoate, and (E)-2-nonenal; 3-methylbutanal with acetic acid and methionol; butanal with 3-methylindole, propanoic acid, homofuraneol, and methionol; ethyl hexanoate with gamma-decalactone; and methional with ammonia.

The discriminating volatile compounds that do not significantly (positive or negative) correlate (i.e., strong null correlation) were 2-methylpropanal and ethyl hexanoate; 3-methylbutanal and hydrogen sulfide; acetic acid and 2,3-butanedione; acetic acid and methyl mercaptan; and between butanal and dimethyl disulfide. Moreover, among the non-discriminating compounds, no significant correlation was observed between butanoic acid and homofuraneol; and diethyl sulfide and methionol. 

Some observations can be inferred from these correlations. For instance, the strong negative correlation between 3-methylbutanoic acid and 3-methylbutanal (both are differentiating compounds) indicates an inverse relationship between these compounds. This is to be expected since 3-methylbutanoic acid is produced from the oxidation of 3-methylbutanal [29,46,47]. Thus, the greater the oxidation of 3-methylbutanal, the more 3-methylbutanoic acid is produced. This relationship could be useful in detecting excessive oxidation in Swiss cheese during ripening. Cheese has a low oxidation/reduction potential, which contributes to the ripening and development of characteristic cheese flavor and ensures limited oxidative degradation [5,48,49]. Excessive oxidation may result in extensive degradation of lipids, primarily polyunsaturated fatty acids. Oxidative degradation of lipids produces various strongly flavored unsaturated aldehydes, resulting in the flavor defect oxidative rancidity [49,50]. One cause of oxidation in cheese is light-induced oxidation due to light exposure during cheese storage and retail distribution [51,52,53,54]. 

A strong negative correlation was also observed between ethyl hexanoate (a discriminating compound) and gamma-decalactone (a non-discriminating compound). Both the ester and lactone are produced from the metabolism of fatty acids during cheese ripening, and these free fatty acid-derived compounds directly affect cheese flavor [44,49]. Esters impart a fruity flavor note (i.e., apple-, banana-, pear-, pineapple-, and strawberry-like) while lactones contribute to a buttery character in cheese [44,49,55]. It is notable that these compounds require different types of glyceride substrate early in their biochemical pathways of production, which are acted upon by different catalytic enzymes from various strains of starter and non-starter lactic acid and propionic acid bacteria [5,43,44]. Triacylglycerides esterified with hydroxylated fatty acid precursor undergo direct lactonization to form lactones, such as gamma-decalactone [43,49,56]. On the other hand, monoglycerides and diglycerides are acted on by lactic acid bacteria esterases to produce esters such as ethyl hexanoate [44,57,58]. Thus, the extent of milk fat hydrolysis to produce mono-, di-, or triacylglycerides, which is in turn influenced by the different lipolytic and esterolytic enzymes, affects the relative formation of these two compounds, which impacts the fruity versus buttery flavor in Swiss cheese [49,59,60]. High levels of ethyl esters cause a fruity flavor defect, while elevated lactone concentration results in high buttery notes in cheese [44,49,58]. 

A strong positive correlation between the two sulfur compounds, dimethyl disulfide (a discriminating compound) and diethyl sulfide (a non-discriminating compound), and butanoic acid (a non-discriminating compound) was observed. The biochemical pathways for these compounds are different, and not obviously connected. The two low molecular weight sulfur compounds are formed predominantly from the elimination reaction-initiated catabolism of the amino acid methionine, so the positive correlation between dimethyl disulfide and diethyl sulfide is expected [61,62,63]. Butanoic acid, on the other hand, is a free fatty acid (FFA) generally produced from the lipolysis of milk fat [5,46]. This positive correlation suggests an implicit association between proteolysis (catabolism of methionine) and lipolysis (generation of FFA) during the ripening of Swiss cheese. This relationship should be further explored to determine whether it is causational or accidental.

A strong positive correlation was also observed between 2,3-butanedione, methyl mercaptan, and hydrogen sulfide (all discriminating compounds). A similarity in the variation of the levels of hydrogen sulfide and methyl mercaptan with the age of cheese has been previously observed [64]. However, there is no clear relationship between the two biochemical pathways, which generate the sulfur-containing compounds from proteolysis, and 2,3-butanedione from citrate metabolism [61,62,65,66]. Thus, this relationship should also be further explored to determine whether it is causational or accidental.

### 3.2. Correlation of Volatile Compounds with Sensory Attributes

Correlations between flavor volatile compounds and sensory attributes were evaluated using Pearson correlation from PCA (Table 4). Not all volatile compounds have strong significant correlations with sensory attributes and vice versa, because volatile compounds may or may not play crucial roles in flavor or contribute to the overall cheese flavor [19,20,48,49,50]. In this study, significant correlations (positive and negative) between volatile compounds (discriminating or non-discriminating) and sensory attributes were observed. These correlations agree with the premise of component balance theory that was first proposed by Kosikowski and Mocquot, which states that a mixture of compounds is responsible for each cheese flavor [12]. Among the discriminating volatile compounds, strongly significant correlations with different sensory attributes (positive attribute, (*negative attribute*); indicated with these fonts) were observed. This includes: 2,3-butanedione with diacetyl and (*sweet*, *sour*, and *umami*); 2-methylpropanal with milkfat lactone, nutty malty, salty; 3-methylbutanal with milkfat lactone; 3-methylbutanoic acid with (*salty*); acetic acid with (*milkfat lactone*); ethyl hexanoate with bitter, cooked cabbage, dried fruit, prickle, sour, sweaty, sweet, umami, vinegar/sour aromatic, and (*cooked milky* and *whey*); and methyl mercaptan with diacetyl and (*umami*). On the other hand, no correlations (i.e., strong null correlation) were observed between 2,3-butanedione with milkfat lactone; 3-methybutanoic acid with dried fruit; and methyl mercaptan with milkfat lactone.

Some notable observations include the significant positive correlation between 2,3-butanedione (a discriminating compound) and the diacetyl sensory attribute, which is expected. Many studies have identified the correlation between 2,3-butanedione and the flavor attribute diacetyl, which is highly associated with a buttery flavor note. Moreover, historically these two terms have been used interchangeably [28,29,67]. 

On the other hand, 2,3-butanedione has a strong negative correlation with the sweet sensory attribute. According to the sensory lexicon in Table 3 and descriptive language of sensory attributes developed by Drake and co-workers [33], this indicates that the fresh butter aroma of 2,3-butanedione is inversely associated with the basic taste sensation elicited by sugars (sweet). It was previously observed that high levels of 2,3-butanedione result in an unbalanced buttery flavor that lacks the sweetness, delicate, and smooth sensation and flavor of fresh milk fat in high-quality butter [68,69]. Thus, based on previous studies, 2,3-butanedione could cause a lower sweetness to be perceived.

Ethyl hexanoate (a discriminating compound) positively correlated with the sensory attributes dried fruit, sweet, and bitter. The positive correlation of ethyl hexanoate with dried fruit and sweet flavor is expected because ester compounds, such as ethyl hexanoate, are known to produce sweet, fruity flavor and are responsible for the aroma of fruits [70,71,72]. Ester compounds were also previously correlated with the sweet odor in Appenzeller, Gruyѐre, Swiss, Italian, and blue cheeses [73]. Similarly, Lawlor and co-workers detected the positive correlation of ester compounds with sweet flavor [67]. The positive correlation between ethyl hexanoate and bitter attribute was unexpected. At low concentration, ethyl hexanoate carries a fruity flavor note in Swiss cheese, however, increased concentrations of ethyl hexanoate and other ethyl esters were strongly associated with fruity flavor defects in Cheddar cheese [7,20,71,74]. It could be that high levels of the fruity flavor of ethyl hexanoate are eliciting the bitter sensory attribute in Swiss cheese. 

Gamma-decalactone positively correlated with cooked milky and diacetyl flavor attributes. Lactones are generally characterized by very pronounced fruity notes, although they have been found to contribute to a buttery character in cheese [49,55]. Thus, it must be the buttery flavor characteristic of gamma-decalactone that was associated with cooked milky and diacetyl flavor notes detected in Swiss cheese.

The alkyl aldehydes 2-methylpropanal and 3-methylbutanal (both are discriminating compounds) have a strong positive correlation with the flavor attributes nutty malty and salty. The correlation of 2-methylpropanal and 3-methylbutanal with nutty malty flavor agrees with previous sensory studies, which reported that the Strecker aldehydes 2-methylpropanal, 2-methylbutanal, and 3-methylbutanal contribute nutty flavors in aged Cheddar cheese [75,76]. Parmesan and Swiss cheese, which are characterized by their intense nutty flavor, have Strecker aldehydes as predominant compounds [77,78,79,80,81,82]. The positive correlation between 3-methylbutanal and salty flavor also agrees with previous sensory studies, particularly in cured ham [83,84,85]. 

**Figure 2:**
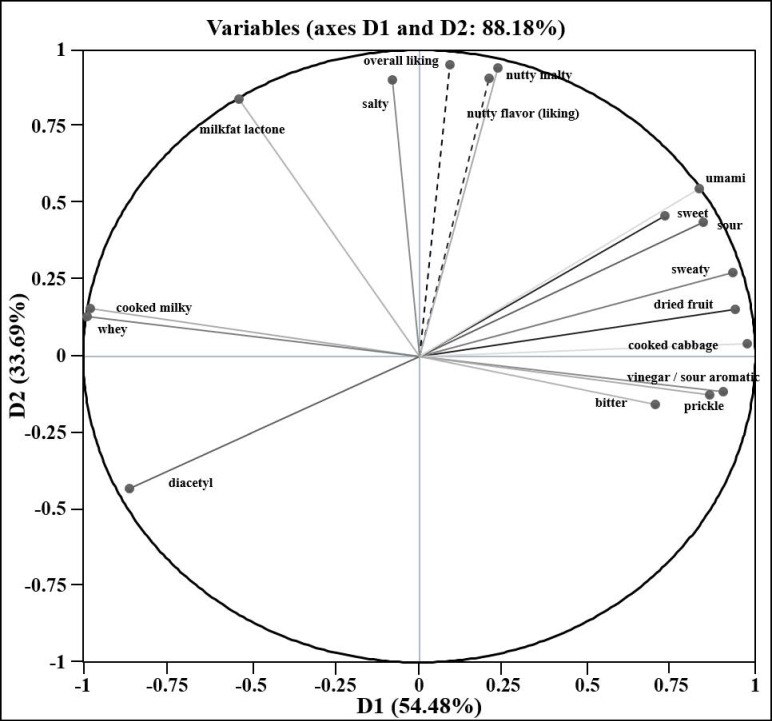
Principal component analysis showing a correlation circle projection of sensory attributes with overall liking and nutty flavor liking.

Consumer liking was also measured for these cheeses with an untrained panel. Correlation analysis (Figure 2) between consumer preference and the descriptive panel sensory attributes showed a positive correlation of five attributes (nutty malty, milkfat lactone, salty, umami, and sweet) with consumer preference (overall liking and nutty flavor liking) (Table 5). Thus, these attributes appeared to be the most important to consumer preference. No sensory attribute had a strongly negative correlation with overall liking or nutty flavor liking, which may be due to the Swiss cheese samples tested not having any strong, noticeable flavor defects. Thus, for good quality Swiss cheese, the sensory attributes nutty malty, milkfat lactone, salty, umami, and sweet could be well-associated with overall liking and nutty flavor liking.

## 4. Conclusions

In this study, flavor variability among Swiss cheeses without perceived flavor defects was evaluated using correlations between volatile compounds and sensory attributes. These correlations brought about further understanding of the complexity of flavor and flavor variability among Swiss cheese samples manufactured from different factories. The important discriminating volatile compounds were classified into five functional groups, namely sulfur-containing compounds (methyl mercaptan, hydrogen sulfide, dimethyl disulfide, dimethyl trisulfide, and methional), organic acids (propanoic acid, acetic acid, 3-methylbutanoic acid), aldehydes (3-methylbutanal, butanal, and 2-methylpropanal), a ketone (2,3-butanedione), and an ester (ethyl hexanoate). The prevailing biochemical activities during the manufacture and ripening of cheese determine the volatile profile of cheese, but the activity of the pathways could be characteristically different for each factory, causing volatile flavor compound variation among Swiss cheeses. 

The correlations between descriptive sensory attributes and volatile compounds showed that only a subset of compounds strongly correlates positively or negatively to a specific attribute. These attribute-volatile compound correlations, albeit partial, not only show the flavor complexity of Swiss cheese but also highly suggest, like many other studies have concluded, that the composition of cheese flavor results from the interaction between several volatile compounds and other compositional variables mixed in different and balanced ratios [73,86,87]. For instance, during lipolysis and metabolism of fatty acids, the extent of the hydrolysis of milk fat directly affects the production of different glyceride substrates, which then affects the production of esters (i.e., requires mono- or diglycerides substrate) versus lactones (i.e., requires triacylglyceride substrate). This could then result in either a more fruity (from high levels of esters) or buttery (from elevated level of lactones) flavor of the Swiss cheese. Correspondingly, during citrate metabolism, a more active 2,3-butanedione production and/or its inhibited metabolism could increase 2,3-butanedione concentration in cheese, which could then intensify the buttery flavor of Swiss cheese but could result in lower perceived sweetness of the cheese.

Finally, the overall liking and nutty flavor liking of Swiss cheese were strongly positively correlated with nutty malty, milkfat lactone, salty, umami, and sweet based on consumer preference evaluation. Thus, for cheeses without flavor defects, future sensory analysis on these five sensory attributes could be evaluated based on the overall liking and nutty flavor liking for a more concise flavor quality evaluation of Swiss cheeses.

## Figures and Tables

**Table 1:** Swiss cheese samples age, starter microorganism, and adjunct culture used by the different factories in Swiss cheese making.

Factory	Cheese Age (Days) at the Time of Packaging (Post-Curing and Ripening)	*Streptococcus (S.)* ^a^	*Propionibacterium* (*P.*) ^a^	Primary *Lactobacillus* (*L.)* ^a^	Adjunct *Lactobacillus (L.)* ^a^
148	30	*S. thermophilus*	*P. freudenreichii*	*L. delbrueckii*	*L. casei*
207	54	*S. thermophilus*	*P. freudenreichii*	*L. helveticus*	*Lactobacillus*
374	35–36	*S. thermophilus*	*P. freudenreichii*	*L. helveticus*	*L. rhamnosus*
465	32	*S. thermophilus*	*P. freudenreichii*	*L. helveticus*	None
528	31	*S. thermophilus*	*P. freudenreichii*	*L. delbrueckii var lactis*	*L. casei*

^a^ Each factory uses different and undisclosed strains for each of their starters and adjunct cultures.

**Table 2:** Selected ion mode method. List of volatile compounds and kinetic information (i.e., reagent ion, rate coefficients, and product ions) used in volatile compound analysis using selected ion flow tube-mass spectrometry (SIFT-MS).

Compound	Reagent	Reaction Rate, *k* (cm^3^/s)	Branching Ratio (%)	Mass-to-Charge Ratio (*m*/*z*)	Product
(E)-2-nonenal	H_3_O^+^	4.8 × 10^−9^	100	141	C_9_H_17_O^+^
	H_3_O^+^			159	C_9_H_17_O^+^•H_2_O
	NO^+^	3.8 × 10^−9^	80	139	C_9_H_15_O^+^
	NO^+^		20	169	C_9_H_15_O^+^•NO^+^
	O_2_^+^	3.7 × 10^−9^	18	83	C_5_H_7_O^+^
				84	C_5_H_8_O^+^
				96	C_7_H_12_^+^
2,3-butanedione	H_3_O^+^	1.7 × 10^−9^	100	87	C_4_H_7_O_2_^+^
	NO^+^	1.3 × 10^−9^	35	43	C_2_H_3_O^+^
	NO^+^		65	86	C_4_H_6_O_2_^+^
2-methylpropanal	O_2_^+^	3.0 × 10^−9^	70	72	C_4_H_8_O^+^
3-methylbutanal	NO^+^	3.0 × 10^−9^	100	85	C_5_H_9_O^+^
	H_3_O^+^	3.6 × 10^−9^	30	65	C_5_H_6_^+^
3-methylbutanoic acid	H_3_O^+^	3.0 × 10^−9^	95	103	C_5_H_11_O_2_^+^
	NO^+^	2.5 × 10^−9^	70	132	C_5_H_10_O_2_^+^•NO^+^
3-methylindole	H_3_O^+^	3.3 × 10^−9^	100	132	C_9_H_10_N^+^
	NO^+^	2.5 × 10^−9^	100	131	C_9_H_10_N^+^
	O_2_^+^	2.4 × 10^−9^	20	130	C_9_H_10_N^+^
			75	131	C_9_H_10_N^+^
acetic acid	H_3_O^+^	2.6 × 10^−9^	100	61	CH_3_COOH_2_^+^
				79	CH_3_COOH_2_^+^•H_2_O
				97	CH_3_COOH_2_^+^•2H_2_O
ammonia	H_3_O^+^	2.6 × 10^−9^	100	18	NH_4_^+^
		2.6 × 10^−9^		36	NH_4_^+^•H_2_O
	O_2_^+^	2.6 × 10^−9^	100	17	NH_3_^+^
butanal	NO^+^	3.5 × 10^−9^	100	71	C_4_H_7_O^+^
butanoic acid	H_3_O^+^	2.9 × 10^−9^	90	89	C_3_H_7_COOH_2_^+^
				107	C_3_H_7_COOH_2_^+^•2H_2_O
	NO^+^	1.9 × 10^−9^	50	118	NO^+^•C_3_H_7_COOH
diethyl sulfide	O_2_^+^	2.5 × 10^−9^	30	75	C_3_H_7_S^+^
		2.5 × 10^−9^	35	90	C_4_H_10_S^+^
dimethyl disulfide	NO^+^	2.4 × 10^−9^	100	94	(CH_3_)_2_S_2_^+^
	O_2_^+^	2.3 × 10^−9^	80	94	(CH_3_)_2_S_2_^+^
dimethyl sulfide	H_3_O^+^	2.5 × 10^−9^	100	63	(CH_3_)_2_S.H^+^
	NO^+^	2.2 × 10^−9^	100	62	(CH_3_)_2_S^+^
	O_2_^+^	2.2 × 10^−9^	25	47	CH_3_S^+^
dimethyl trisulfide	H_3_O^+^	2.8 × 10^−9^	100	127	C_2_H_6_S_3_H^+^
	NO^+^	1.9 × 10^−9^	100	126	C_2_H_6_S_3_^+^
	O_2_^+^	2.2 × 10^−9^	15	111	CH_3_S_3_^+^
	O_2_^+^	2.2 × 10^−9^	45	126	C_2_H_6_S_3_^+^
ethanol	H_3_O^+^	2.7 × 10^−9^	100	47	C_2_H_7_O^+^
		2.7 × 10^−9^		65	C_2_H_7_O^+^•H_2_O
		2.7 × 10^−9^		83	C_2_H_7_O^+^•(H_2_O)_2_
	NO^+^	1.2 × 10^−9^	100	45	C_2_H_5_O^+^
		1.2 × 10^−9^		63	C_2_H_5_O^+^•H_2_O
		1.2 × 10^−9^		81	C_2_H_5_O^+^•(H_2_O)_2_
	O_2_^+^	2.3 × 10^−9^	75	45	C_2_H_5_O^+^
		2.3 × 10^−9^		63	C_2_H_5_O^+^•H_2_O
		2.3 × 10^−9^		81	C_2_H_5_O^+^•2H_2_O
ethyl butanoate	H_3_O^+^	3.0 × 10^−9^	80	117	C_6_H_12_O_2_•H^+^
				135	C_6_H_13_O_2_^+^•H_2_O
	NO^+^	2.4 × 10^−9^	30	146	C_6_H_12_O_2_^+^•NO^+^
ethyl hexanoate	H_3_O^+^	3.0 × 10^−9^	100	145	C_8_H_16_O_2_•H^+^
	H_3_O^+^	3.0 × 10^−9^		163	C_8_H_16_O_2_•H^+^•H_2_O
	NO^+^	2.5 × 10^−9^	95	174	C_8_H_16_O_2_^+^•NO^+^
ethyl methyl sulfide	H_3_O^+^	2.4 × 10^−9^	100	77	CH_3_SHC_2_H_5_^+^
furaneol	H_3_O^+^	4.0 × 10^−9^	100	129	C_6_H_8_O_3_•H^+^
		4.0 × 10^−9^		147	C_6_H_8_O_3_•H_3_O^+^
	NO^+^	2.5 × 10^−9^	95	128	C_6_H_8_O_3_^+^
	O_2_^+^	3.0 × 10^−9^	100	128	C_6_H_8_O_3_^+^
γ-decalactone	H_3_O^+^	3.0 × 10^−9^	100	171	C_10_H_18_O_2_•H^+^
	NO^+^	2.5 × 10^−9^	100	200	C_10_H_18_O_2_•NO^+^
homofuraneol	H_3_O^+^	3.0 × 10^−9^	100	143	C_7_H_10_O_3_•H^+^
	H_3_O^+^	3.0 × 10^−9^		161	C_7_H_10_O_3_•H^+^•H_2_O
	NO^+^	2.5 × 10^−9^	100	142	C_7_H_10_O_3_^+^
	O_2_^+^	2.5 × 10^−9^	100	142	C_7_H_10_O_3_^+^
hydrogen sulfide	H_3_O^+^	1.6 × 10^−9^	100	35	H_3_S^+^
		1.6 × 10^−9^		53	H_3_S^+^•H_2_O
	O_2_^+^	1.4 × 10^−9^	100	34	H_2_S^+^
methional	O_2_^+^	2.5 × 10^−9^	75	104	C_4_H_8_OS^+^
methionol	NO^+^	2.5 × 10^−9^	100	106	C_4_H_10_OS^+^
	O_2_^+^	2.5 × 10^−9^	30	89	C_4_H_9_S^+^
		2.5 × 10^−9^	40	106	C_4_H_10_OS^+^
methyl mercaptan (methanethiol)	H_3_O^+^	1.8 × 10^−9^	100	49	CH_4_S.H^+^
		1.8 × 10^−9^		67	CH_4_S.H^+^•H_2_O
phenylacetaldehyde	H_3_O^+^	3.0 × 10^−9^	100	121	C_8_H_8_O•H^+^
	H_3_O^+^	3.0 × 10^−9^		157	C_8_H_8_O•H^+^•2H_2_O^+^
	NO^+^	2.5 × 10^−9^	15	91	C_7_H_7_^+^
	NO^+^	2.5 × 10^−9^	60	120	C_8_H_8_O^+^
	NO^+^	2.5 × 10^−9^	25	150	C_8_H_8_O•NO^+^
	O_2_^+^	2.5 × 10^−9^	40	91	C_7_H_7_^+^
	O_2_^+^	2.5 × 10^−9^	40	92	C_7_H_8_^+^
	O_2_^+^	2.5 × 10^−9^	20	120	C_8_H_8_^+^
	O_2_^+^	2.5 × 10^−9^		121	C_8_H_8_O•H^+^
propanoic acid	H_3_O^+^	2.7 × 10^−9^	90	75	C_2_H_5_COOH_2_^+^
	NO^+^	1.5 × 10^−9^	30	57	C_2_H_5_CO^+^
	O_2_^+^	2.2 × 10^−9^	80	74	C_2_H_5_COOH^+^
tetramethylpyrazine	H_3_O^+^	3.0 × 10^−9^	100	137	C_8_H_12_N_2_•H^+^
	NO^+^	2.5 × 10^−9^	100	136	C_8_H_12_N_2_^+^
	O_2_^+^	2.5 × 10^−9^	100	136	C_8_H_12_N_2_^+^

**Table 3:** Swiss cheese descriptive analysis lexicon, adapted from [30,33].

Swiss Cheeses Sensory Descriptor	Definition
Bitter	Fundamental taste sensation elicited by various compounds
Cooked cabbage	Aromatics associated with cooked cabbage
Cooked/milky	Aromatics associated with cooked milk
Diacetyl (buttery)	Aromatics associated with diacetyl
Dried fruit	Aromatics associated with dried fruits, specifically peaches and apricots
Milk fat	Aromatics associated with milk fat
Nutty	Nutlike aromatic associated with different nuts
Prickle	Chemical feeling factor of which the sensation of carbonation on the tongue is typical
Salty	Fundamental taste sensation elicited by salts
Sour	Fundamental taste sensation elicited by acids
Sweaty	Aromatic associated with human sweat
Sweet	Fundamental taste sensation elicited by sugars
Umami	Chemical feeling factor elicited by certain peptides and nucleotides
Vinegar	Aromatics associated with vinegar
Whey	Aromatics associated with Cheddar cheese whey

**Table 4:** Pearson correlation between sensory attributes and volatile compounds.

Sensory Attribute	Compounds with Positive Correlation		Compounds with Negative Correlation		Sensory Attribute	Compounds with Positive Correlation		Compounds with Negative Correlation	
	Compound	Score, *r*	Compound	Score, *r*		Compound	Score, *r*	Compound	Score, *r*
**bitter**	ethyl hexanoate	0.87	furaneol	−0.90	**sweaty**	ethyl hexanoate	0.78	ethanol	−0.94
	dimethyl sulfide	0.65	diethyl sulfide	−0.82		homofuraneol	0.78	gamma-decalactone	−0.93
			butanoic acid	−0.79		propanoic acid	0.74	2,3-butanedione	−0.89
			butanal	−0.73				methyl mercaptan	−0.89
			gamma-decalactone	−0.71				hydrogen sulfide	−0.83
**cooked cabbage**	ethyl hexanoate	0.96	gamma-decalactone	−0.98				tetramethylpyrazine	−0.78
	propanoic acid	0.80	furaneol	−0.87				furaneol	−0.74
	homofuraneol	0.78	ethanol	−0.85				dimethyl disulfide	−0.71
			methyl mercaptan	−0.79	**sweet**	ethyl methyl sulfide	0.79	ethanol	−0.93
			2,3-butanedione	−0.79		3-methylindole	0.73	methyl mercaptan	−0.90
			butanal	−0.77		ethyl hexanoate	0.71	hydrogen sulfide	−0.88
			tetramethylpyrazine	−0.75		propanoic acid	0.71	2,3-butanedione	−0.87
			hydrogen sulfide	−0.71				dimethyl trisulfide	−0.79
**cooked milky**	gamma-decalactone	0.98	ethyl hexanoate	−0.96				tetramethylpyrazine	−0.77
	furaneol	0.85	homofuraneol	−0.76				gamma-decalactone	−0.75
	butanal	0.81	propanoic acid	−0.69				furaneol	−0.72
	2-methylpropanal	0.71			**umami**	ethyl hexanoate	0.70	2,3-butanedione	−0.99
**diacetyl**	2,3-butanedione	0.93	ethyl hexanoate	−0.68		homofuraneol	0.50	methyl mercaptan	−0.98
	ethanol	0.91	homofuraneol	−0.59				ethanol	−0.97
	methyl mercaptan	0.91						hydrogen sulfide	−0.96
	hydrogen sulfide	0.87						tetramethylpyrazine	−0.94
	tetramethylpyrazine	0.87						dimethyl disulfide	−0.91
	gamma-decalactone	0.86						gamma-decalactone	−0.84
	dimethyl disulfide	0.85						furaneol	−0.81
	furaneol	0.74						methional	−0.75
**dried fruit**	ethyl hexanoate	0.77	gamma-decalactone	−0.93				dimethyl trisulfide	−0.75
	homofuraneol	0.76	ethanol	−0.83	**vinegar/sour aromatic**	propanoic acid	0.93	gamma-decalactone	−0.91
	propanoic acid	0.63	2,3-butanedione	−0.81		ethyl hexanoate	0.90	butanal	−0.85
			methyl mercaptan	−0.79		homofuraneol	0.87	ethanol	−0.75
			tetramethylpyrazine	−0.74		methionol	0.79	2-methylpropanal	−0.72
			hydrogen sulfide	−0.72		3-methylindole	0.72	furaneol	−0.72
			furaneol	−0.72	**whey**	gamma-decalactone	0.99	ethyl hexanoate	−0.95
**milkfat lactone**	2-methylpropanal	0.93	(E)-2-nonenal	−0.85		butanal	0.82	homofuraneol	−0.85
	3-methylbutanal	0.84	3-methylbutanoic acid	−0.69		furaneol	0.80	propanoic acid	−0.81
	butanal	0.84	ethyl butanoate	−0.66		ethanol	0.75	methionol	−0.62
			homofuraneol	−0.63		2-methylpropanal	0.71		
**nutty malty**	3-methylbutanal	0.94	dimethyl disulfide	−0.94		2,3-butanedione	0.69		
	2-methylpropanal	0.83	ethyl butanoate	−0.93		methyl mercaptan	0.68		
			3-methylbutanoic acid	−0.83		tetramethylpyrazine	0.64		
			hydrogen sulfide	−0.82					
			2,3-butanedione	−0.77					
			methyl mercaptan	−0.76					
			methional	−0.74					
			tetramethylpyrazine	−0.72					
**prickle**	ethyl hexanoate	0.77	gamma-decalactone	−0.85					
	homofuraneol	0.58	diethyl sulfide	−0.83					
			butanoic acid	−0.79					
			furaneol	−0.73					
**salty**	3-methylbutanal	0.94	ethyl butanoate	−0.98					
	2-methylpropanal	0.82	3-methylbutanoic acid	−0.76					
	butanal	0.70	dimethyl disulfide	−0.74					
**sour**	homofuraneol	0.68	ethanol	−0.94					
	ethyl hexanoate	0.65	2,3-butanedione	−0.92					
	propanoic acid	0.61	methyl mercaptan	−0.91					
			hydrogen sulfide	−0.88					
			gamma-decalactone	−0.84					
			dimethyl disulfide	−0.81					
			tetramethylpyrazine	−0.80					

**Table 5:** Pearson correlation between the overall consumer preference (overall liking and nutty flavor liking) with sensory attributes.

Overall Consumer Preference	Sensory Attributes with Positive Correlation	
	Attribute	Score, *r*
**overall liking**	nutty malty	0.86
	milkfat lactone	0.75
	salty	0.73
	umami	0.58
	sweet	0.56
**nutty flavor (liking)**	nutty malty	0.85
	umami	0.66
	salty	0.66
	milkfat lactone	0.64
	sweet	0.58
